# U.S. EPA’s Toxicity Reference Database: Martin and Dix Respond

**DOI:** 10.1289/ehp.0900951R

**Published:** 2009-10

**Authors:** Matthew T. Martin, David J. Dix

**Affiliations:** National Center for Computational Toxicology, Office of Research & Development, U.S. Environmental Protection Agency, Research Triangle Park, North Carolina, E-mail:Martin.Matt@epamail.epa.gov

We appreciate the letter from Janus of CropLife America commenting on that group’s assessment of the database and our article (Martin et al. 2009a) from its perspective as an agriculture and pest-management trade organization. We also appreciate the CropLife America’s continued interest in the U.S. EPA’s (U.S. Environmental Protection Agency) ToxRefDB and ToxCast research programs, including the review of much of the data entered into ToxRefDB. However, Janus’s comments do not address the ToxRefDB applications presented in our article, but instead create hypothetical uses of the database and reported data.

For example, in Table 3 of our article (Martin et al. 2009a), we presented multi-gender, multisite, and multispecies rodent tumorigens in order to provide data in a systematic and computable format for predictive toxicity models incorporating potency values. In contrast, Janus and CropLife America refer to a hypothetical regulatory application of this same animal tumorigenicity data in a ranking system never suggested in our article.

The U.S. EPA has gone to great lengths to make ToxRefDB and its development as transparent as possible. Three manuscripts and data sets have been published to date (Knudsen et al. 2009; Martin et al. 2009a, 2009b), and the standardized vocabulary and a version of the database are available on the ToxRefDB website (U.S. EPA 2008b). We will continue to make every effort to publicly release information from ToxRefDB as it continues to develop.

We recognize the complexity of pathologic progression to cancer. The end point progression scheme we presented (Martin et al. 2009a) included aggregation of prolifera tive, preneoplastic, and neoplastic lesions for the development of predictive signatures from *in vitro* data coming from the ToxCast research program (U.S. EPA 2008a). This approach is not controversial in the context of predictive toxicology research and is supported by the literature (Cohen and Arnold 2008; Hanahan and Weinberg 2000).

We agree that it is important to incorporate pharmacokinetics and metabolism, including chemical detoxification and activation, into predictive toxicology efforts. However, this issue is outside the scope of our article (Martin et al. 2009a) and is being addressed in other aspects of the ToxCast research program.

Two additional papers on multigenerational reproductive and prenatal developmental toxicity studies in ToxRefDB have been recently published (Knudsen et al. 2009; Martin et al. 2009b), again with the primary goal of providing diverse end points for predictive modeling as part of the ToxCast research program (Dix et al. 2007). Of toxicity end points in ToxRefDB, we are using only those of sufficient quality for predictive modeling, and we are taking care to distinguish between missing versus negative data.

We view ToxRefDB as a valuable resource to the scientific community and one in which the U.S. EPA, stakeholders, and other interested parties can work together to ensure the success of ToxRefDB and the larger ToxCast effort.
